# Upcycling Waste Streams from a Biorefinery Process—A Case Study on Cadmium and Lead Biosorption by Two Types of Biopolymer Post-Extraction Biomass

**DOI:** 10.3390/molecules28176345

**Published:** 2023-08-30

**Authors:** Jarosław Chwastowski, Maciej Guzik, Szczepan Bednarz, Paweł Staroń

**Affiliations:** 1 Department of Engineering and Chemical Technology, Kraków University of Technology, 24 Warszawska St., 31-155 Kraków, Poland; szczepan.bednarz@pk.edu.pl (S.B.); pawel.staron@pk.edu.pl (P.S.); 2 Jerzy Haber Institute of Catalysis and Surface Chemistry, Polish Academy of Sciences, Niezapominajek 8, 30-239 Kraków, Poland; maciej.guzik@ikifp.edu.pl

**Keywords:** spent biomass, bacteria, biosorption, waste reuse, circular economy

## Abstract

This study investigated the possibility of using the spent kind of biomass of *Pseudomonas putida* CA-3 and *Zobelella denitrificans* MW1 obtained after the pilot-scale production of polyhydroxyalkanoates (PHAs) as a biosorbent for the bioremediation of aqueous solutions containing toxic cadmium and lead ions. The material was characterized by means of scanning electron microscopy, Fourier-transformed infrared spectroscopy, nuclear magnetic resonance spectroscopy and amino acid profiling. To check the sorption capacity of spent biomass against Pb and Cd ions, equilibrium studies were performed. To learn about the nature of the sorption process, kinetic modelling was carried out and the obtained results showed that the adsorption process is best described by the pseudo-second-order kinetic model (PSO), which suggests that the sorption process is connected with the chemical bonding of the ions on the sorbent surface. Information provided by the amino acid profile made it possible to predict the adsorption mechanism and FTIR analysis proved the participation of different chemical groups in the removal process. According to the equilibrium studies, the best-fitted isotherm was the Freundlich model for all used materials and metal ions considering the correlation coefficient. Summarizing the results, the spent biomass after the PHA production is an effective biosorbent and can be reused for heavy metal bioremediation.

## 1. Introduction

Lead and cadmium ions are highly toxic heavy metals that present substantial risks to both human health and the environment. These metals are commonly encountered in industrial processes as well as in contaminated soil and water sources [1]. By comprehending sorption behaviors and mechanisms, researchers and environmentalists can work towards mitigating the adverse effects of these ions’ exposure, safeguarding human health, and preserving the ecosystem.

Lead is an extremely poisonous metal that can cause severe harm to various organs in the human body, including the brain. It poses a particular danger to children, potentially impairing cognitive development and leading to learning disabilities and behavioral issues [2]. Exposure to lead can occur through the ingestion of contaminated food or water, inhalation of lead-containing dust or fumes, or occupational exposure in industries such as battery manufacturing or smelting. Similarly, cadmium is a toxic heavy metal that can have detrimental effects on human health, primarily targeting the kidneys and potentially resulting in kidney damage, impaired kidney function, or even kidney failure. Cadmium exposure has also been associated with lung damage, cardiovascular diseases, and certain types of cancer. Inhalation of cadmium-containing fumes, ingestion of contaminated food or water, and exposure in industries such as mining, battery production, and metal plating can all contribute to cadmium poisoning [3,4]. The toxicity of lead and cadmium ions presents significant risks to both human health and the environment.

Sorption studies play a pivotal role in comprehending and mitigating the toxic effects of lead ions. They are a vital component of environmental remediation, as they aid in the removal and immobilization of heavy metals from contaminated sites [5,6,7]. Sorption studies involve the investigation of the sorption behavior of lead and cadmium ions onto various materials, including activated carbon, zeolites, clay minerals, and biomass. Nicola R. et al. used prepared silica-coated magnetic nanocomposites for the removal of toxic lead ions from aqueous solutions [8] and Basel A. et al. in their work used synthesized nanosilica for the removal of Cd and Cu ions [9].

Spent biomass, referring to organic waste materials such as industrial residues or agricultural byproducts, has gained considerable attention as a potential sorbent for removing heavy metals. Biomass materials like plant residues, agricultural byproducts, and biochar have demonstrated promising adsorption capacities for lead and cadmium ions [10,11]. These materials possess large surface areas and contain functional groups that can interact with heavy metal ions through chemical and physical sorption processes. The utilization of spent biomass not only provides a cost-effective and environmentally friendly solution for heavy metal removal but also contributes to the sustainable management of waste materials.

Sorption investigations play a pivotal role, particularly in harnessing spent biomass as a sorbent, for the advancement of effective strategies aimed at remediating sites contaminated with heavy metals. This research centers on the application of spent biomass originating from a biorefinery process. The spotlight is increasingly on bio-based industries nowadays, as novel processes emerge to supplant petroleum-derived products. Our recent achievement involves a canola oil biorefining method resulting in the production of two bacterial polymers, namely polyhydroxy-alkanoates (PHAs) [12]. The fermentation process yields highly dense bacterial cultures containing PHAs, which are then extracted during downstream procedures. This not only yields pure PHA polymer and recoverable solvents, but also yields dry spent biomass. Embracing the principles of a circular economy, all streams generated within such a biorefinery should be valorized. Hence, the concept was born to assess whether this material could function as a biosorbent for lead and cadmium. Our investigation delved into the sorption attributes concerning Cd^2+^ and Pb^2+^ within this by-product of the biorefinery. We analyzed the chemical composition of the biowastes and conducted thorough batch sorption experiments. The innovation of our work lies in employing actual post-production spent biomass, available in substantial quantities after semi-industrial scale operations, as a biosorbent for representative heavy metals.

## 2. Results and Discussion

### 2.1. Spent Biomass Characterisation

Table 1 lists the main parameters of spent microbial biomass used in our studies. As can be seen, the content of nitrogen, phosphorus and mineral components is typical for such materials [13]. It is important to mention that the biowastes contain 20–30 wt.% of the water-soluble fraction containing presumably proteins, sugars and other low-molecular-weight compounds. We have found that despite treatment with non-polar solvents (see Experimental part), the biomass still contains components that are soluble in acetone in amounts of 4–6 wt.%. The results of both FTIR and ^1^H-NMR analyses of the extracts (Figures S1–S3) indicate that the main components of the fractions are glycerol and fatty acids for ZD and PP, respectively, which are residues originating from the growing medium. Since the presence of fatty acids makes the materials more hydrophobic and therefore more difficult to wet by water, we decide to investigate both the raw (ZDR, PPR) as well as acetone-treated biomass samples (ZDE, PPE) to study the effect of those contaminations on metal sorption process parameters.

**Figure 1:**
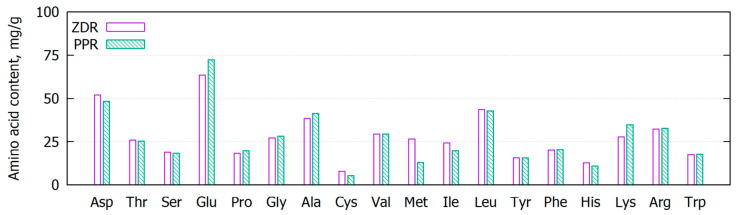
Amino acid composition of the investigated spent bacterial biomass ZDR and PPR.

As proteins are the main components of the dry mass of bacteria [14] and it is well known that they participate in metal sorption processes, we also analyzed the amino acid composition of the spent biomass (Figure 1). As can be seen, the amino acid profile of the two samples is very similar and typical for whole-cell microbial biomass [15]. The total amount of amino acids in the materials is approximately 500 mg/g, and aspartic and glutamic acid are the dominant amino acids, while cysteine is the lowest. In addition to the aforementioned amino acids with carboxyl side groups, the biomass contains basic amino acids, which are also able to participate in the metal-binding process, i.e., arginine, lysine, and histidine [16].

### 2.2. Metal Sorption Studies

**Figure 2:**
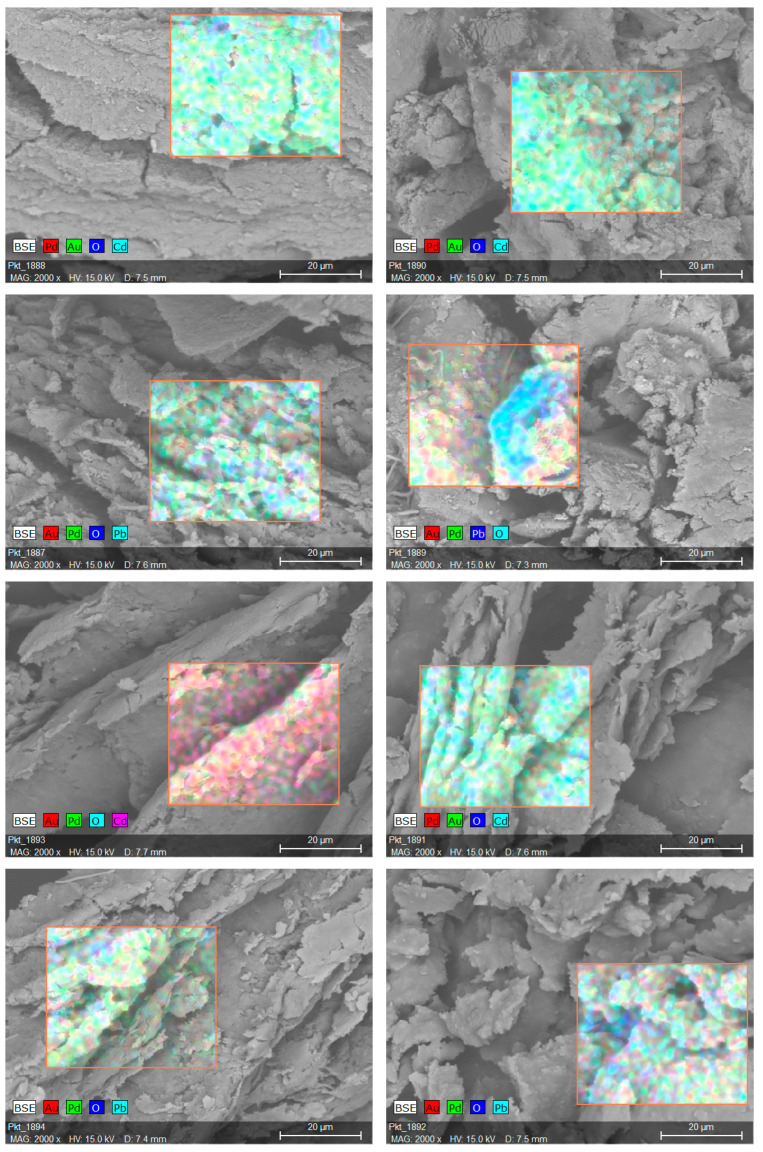
SEM-EDX microphotographs of ZDR-Cd, ZDE-Cd, ZDR-Pb, ZDE-Pb, PPR-Cd, PPE-Cd, PPR-Pb, and PPE-Pb, respectively.

In preliminary studies, four materials ZDE, ZDR, PPE and PPR were dispersed in an aqueous solution of Cd^2+^ and Pb^2+^ salts, then filtered out, dried and analyzed using scanning electron microscopy. One can see that the surface is rather heterogeneous with varied structures (from plain areas to large pores) with all the used materials. This kind of morphology allows for the absorption of higher amounts of metal ions due to the higher surface area compared to materials with low porosity. It can be seen that biomass does not have a spheroidal or bacterial-like shape. This phenomenon is due to the downstream processing of the harvested biomass, leading to cell death and disruption. The result of the EDX analysis of all samples after both cadmium and lead removal experiments is shown in Figure 2. The analysis proved qualitatively that the lead and cadmium ions were captured from the aqueous solution and sorbed on the biomass surface. The results prompted us to carry out more detailed and quantitative sorption studies.

### 2.3. Point of Zero Charge (PZC)

**Figure 3:**
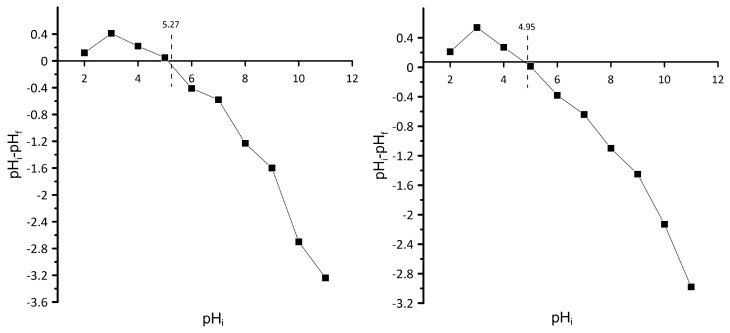
Point of zero charge for spent biomass.

To check the best pH conditions for the maximal sorption capacity and optimal removal efficiency of metal ions of obtained spent biomass, the point of zero charge PZC has been calculated for both ZD and PP spent biomass. According to the literature, the adsorption properties of sorbents are influenced by the PZC, which is the pH at which the adsorbent surface charge is equal to 0. The shift in pH of the aqueous dispersion is plotted against the initial pH. The point at which the plot crosses through 0 is named the PZC. In this study, the PZC was determined to be equal to 4.95 for ZD and 5.27 for PP, which follows the literature data [17] (Figure 3). Due to the electrostatic attraction between negative charges on the spent biomass surface and positively charged cadmium and lead cations, the occurrence of the sorption above pH equal to 4.95 for ZD and 5.27 for PP is more favorable. In more acidic conditions, there would be competition for active binding sites on the sorbent between hydrogen and metal ions, overall decreasing the sorption capacity of the material.

### 2.4. Isotherm and Kinetics

**Figure 4:**
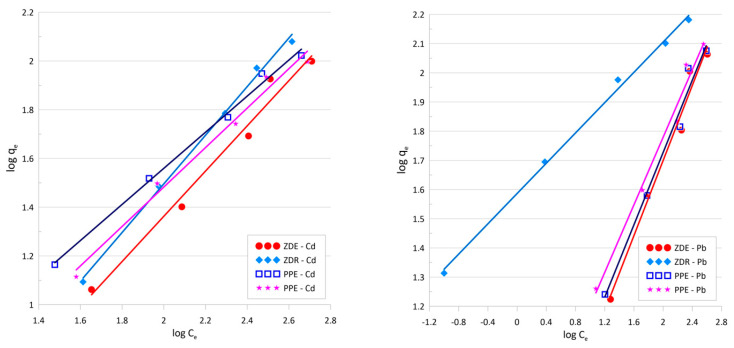
Freundlich isotherm model for Cd and Pb, respectively.

Table 2 and Figure 4 presents the results obtained for the Freundlich isotherm model. According to the correlation coefficient, the R^2^ Freundlich isotherm shows the best fit for all of the investigated materials and metal ions (the values obtained for the other tested models are in the Supplementary Material). The Freundlich isotherm presumes the adsorption on the heterogeneous surface is valid only up to a certain concentration, which is a limiting step in the process. Additionally, the value of the 1/n parameter calculated from the Freundlich isotherm is below 1, which implies that the process is based on the chemical reaction between the ions and the sorbent surface.

In Table 3, one can see the parameters obtained for the PSO model (pseudo-second-order model), which has the highest correlation coefficient compared to other models (see Supplementary Materials).

**Figure 5:**
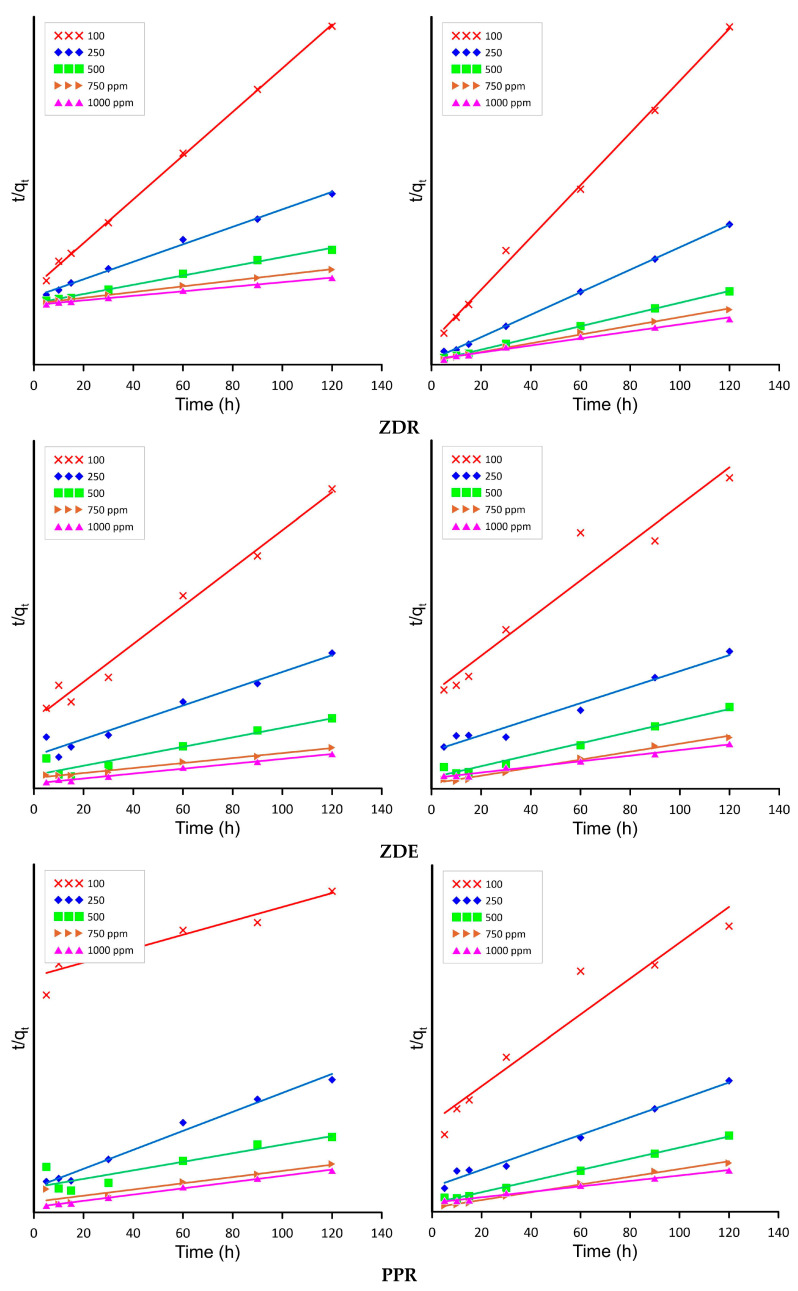

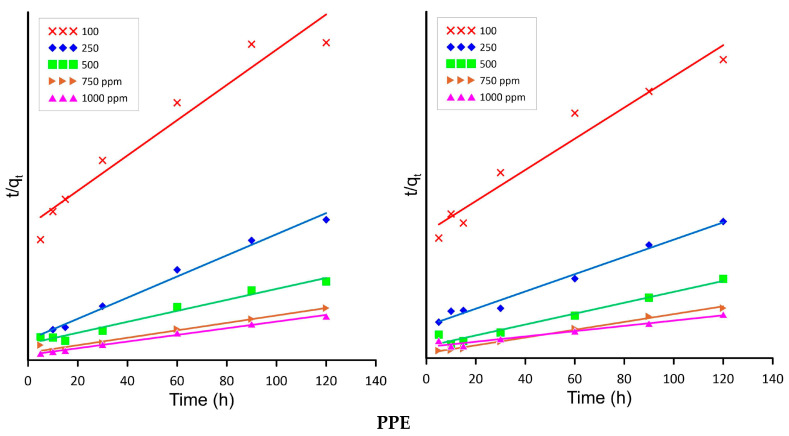
(**ZDR**), (**ZDE**), (**PPR**), (**PPE**) pseudo-second kinetic model for Cd and Pb adsorption, respectively.

Figure 5 shows the graphs of the PSO kinetic model for all the used concentrations of 100, 250, 500, 750, and 1000 ppm of both Cd^2+^ and Pb^2+^ for ZDR, ZDE, PPR and PPE biomass.

The performed calculations have led to the conclusion that the model that best describes the bioremediation process of both lead and cadmium ions on all the tested spent biomass is the PSO kinetic model. The average correlation coefficient is at its highest across all the tested concentrations, and the values of the average relative error (ARE) are consistently below 1%. The rate-limiting step in this type of adsorption is connected with surface adsorption, which includes the chemical binding of metal ions that is proved inter alia by the changes in the FTIR spectra before and after the sorption process (Figure 5). The obtained results confirming the nature of sorption with the use of spent biomass are similar to those obtained by Ramrakhiani L. et al. [18].

The physicochemical interactions between sorbate and the adsorbent result in the binding of the metal ions on the surface of biomass. Additionally, the lower the k_2_ constant, the higher the affinity of the adsorbent to lead ions occurs. Comparing the obtained results of the k_2_ constant to those reported by [19], the values are low, suggesting that strong, fast metal ion sorption with favorable conditions between spent biomass and metal ions takes place. The calculated q_e_ values from PSO are similar to those obtained from the experimental data, suggesting consistency with a chosen kinetic model. The results obtained by other researchers are similar. For example, Mohammed Nasser (2018) presented the performance of *Streptomyces rimosus* biomass in the biosorption of heavy metals from aqueous solutions, where the pseudo-second-order rate model also demonstrated the best fit [20].

### 2.5. Sorption Mechanism

In this study, we investigated the use of non-living Gram-negative bacterial cell biomass as heavy metal cation sorbents. As expected, the chemical composition of these biological materials was very similar. The only difference we observed was the presence of culture medium residues, i.e., glycerol and fatty acids for ZD and PP, respectively. Both species, i.e., ZD and PP, form rod-shaped cells with a size of c.a. 1.0 µm in length but after freezing, lyophilization and PHA extraction, the cells were disrupted and formed irregular fine particles. It is known that the main pathways for metal binding include complexation, ion exchange and microprecipitation [21]. The processes could be limited by physical phenomena such as mass transfer, diffusion through the liquid layer, diffusion into pores etc. Both chemical and physical factors could impact on metal sorption mechanism, thus the process kinetics.

Keeping in mind that the investigated biomass is rich in amino acids, i.e., aspartic and glutamic acid (Figure 1), it could be hypothesized that the proteins play the main role in complexation and ion exchange processes, leading to the binding of Cd^2+^ and Pb^2+^ ions. As described in the Experimental part, the downstream processing of the investigated biomass is focused on the effective extraction of the biopolymers from the bacterial cells using non-polar solvents. In this way, the biomass contains the initial amount of water-soluble substances, including proteins, peptides, and amino acids, which may also participate in heavy metal binding via the microprecipitation mechanism [22].

The culture medium components (glycerol and fatty acids), as well as possible PHA residues, may impact the physical form of the dry spent biomass, and thus influence sorption properties via physical factors. As can be seen on the SEM images (Figure 2), the bacterial particles are agglomerated into larger debris and form a greasy powder observed on a macro scale. In the case of ZDR samples, it seems that the presence of glycerol as contamination, which is miscible with water and does not form chelates with Cd^2+^ and Pb^2+^ cations, is not relevant for the metal sorption processes. In contrast, fatty acid residues present in PPR biomass increase the hydrophobicity of this material while reducing wettability. This phenomenon may impact the kinetics of metal sorption as it causes pore blocking and thus increases diffusion resistance.

**Figure 6:**
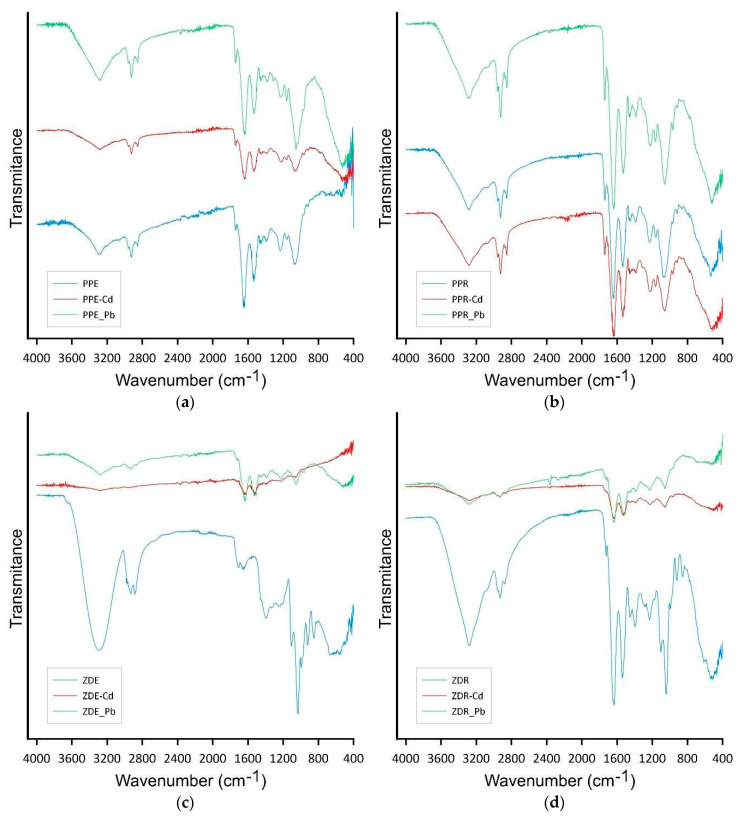
FTIR analysis of extract obtained from spent biomass after washing with acetone (**a**), PPE before and after sorption (**b**), PPR before and after sorption (**c**), ZDE before and after sorption (**d**) ZDR before and after sorption.

The FTIR spectra of the samples presented in Figure 6 reveal some differences, particularly in the region 1730–1300 cm^−1^. These bands are commonly associated with the functional groups of polyester from the polyhydroxyalkanoate series. For example, in *azospirilla,* these polyesters are primarily represented by the homopolymer poly-3-hydroxybutyrate (PHB), as documented in Kamnev et al. 2008 [23]. Variations in the intensity of inter- and intramolecular interactions formed by these hydrogen bonds, particularly between the ester carbonyl group (which exhibits a band at approximately 1720–1750 cm^−1^ due to C=O stretching vibrations, sensitive to H-bonding) and the –CH_3_ group in the polymer chains, resulting in variability in the degree of ordering or crystallinity. The samples of spent bacteria cells show in the region 1645–1550 cm^−1^ the amid band activity. The band between 3700 and 2700 cm^−1^ is connected with stretching vibration of O–H and N–H groups, and 1485–1000 cm^−1^ (C–O, C–C, C–O–H, C–O–C band from polysaccharides and polyesters). The changes in the peaks intensity after the sorption process suggest that different groups participate in the process of metal ion removal, thus suggesting and confirming the chemical nature of the adsorption.

### 2.6. Desorption Studies

The reusability of spent biomass was studied with the use of different 0.1 M organic acids, mineral salts and additionally, deionized water as a control. In Table 4, the results of three cycles are shown. One can see that the desorption process is strongly connected with the used tyle of eluent. Citric acid has the highest removal efficiency for both metal ions in accordance with the literature [24] The mechanism is probably connected with the formation of complexes due to the chelation process. No desorption of ions with the use of water was observed, indicating a strong chemical bonding between the metal ions and the sorbent surface.

## 3. Materials and Methods

### 3.1. Chemicals

The reagents were purchased from MERCK (Darmstad, Germany) and were of analytical purity. All metal salt solutions were prepared in deionized water.

### 3.2. General Methods

To acquire information about the surface morphology of spent biomass before sorption and after the Pb and Cd ion removal process, a high-resolution electron scanning microscope (Hitachi TM-3000, Tokyo, Japan) equipped with an X-ray dispersion spectrometer was used. The biomass was also characterized by means of the FTIR-ATR method using a Thermo Scientific (Waltham, MA, USA) Nicolet iS5 spectrometer with the ATR iD7 attachment. Each sample was measured over a wavenumber in the range between 400 and 4000 cm^−1^ with a resolution of 0.5 cm^−1^. The metal cation concentrations were measured directly in solutions after filtration of biomass, employing AAS (atomic absorption spectrophotometry) in the ionized flame (Perkin-Elmer AAS, Waltham, MA, USA).

### 3.3. Spent Biomass

The two kinds of biomass were obtained in a biorefinery process for the conversion of rapeseed oil derivatives (glycerol and fatty acids) into two types of biodegradable polyhydroxyalkanoate polymers, namely polyhydroxybutyrate [P(3HB)] and medium-chain-length polyhydroxyalkanoate (mcl-PHA), respectively. Both PHAs were obtained in a semi-industrial, pilot-scale biorefinery prototype in a 200 L bioreactor (Biostat D, Sartorius Stedim, Göttingen, Germany). *Zobellella denitrificans* MW1 was used to obtain P(3HB) with glycerol as a substrate according to a scaled-up protocol in mineral salt medium 1 (MSM1) that contained (g/L): Na_2_HPO_4_ × 12H_2_O, 9.0; KH_2_PO_4_, 1.5; MgSO_4_ × 7H_2_O, 0.2; NH_4_Cl, 1.0; NaCl, 15.0; CaCl_2_ × 2H_2_O, 0.02; and Fe(III)NH_4_^+^-citrate, 0.0012. The MSM1 also included 1 mL of trace element solution in 1M HCl containing the following (g/L): FeCl_3_, 8.3; ZnCl_2_, 0.84; CuCl_2_ × 2H_2_O, 0.13; CoCl_2_ × 6H_2_O, 0.1; MnCl_2_ × 6H_2_O, 0.016; and H_3_BO_3_, 0.1. The second process employed *Pseudomonas putida* CA-3 to produce mcl-PHA polymers from rapeseed oil fatty acids in mineral salt medium 2 (MSM2) that contained (g/L): (NH_4_)_2_SO_4_, 4.7; MgSO_4_ × 7H_2_O, 3.0; Na_2_HPO_4_ × 7H_2_O, 15.0; KH_2_PO_4_, 3.40 and trace elements. Downstream processing for both fermentations was similar. Briefly, after each bioprocess, the biomass was separated by centrifugation from the fermentation broth, frozen and freeze-dried. The polymers were extracted with the use of organic solvents, including P(3HB) with the aid of chloroform. The mcl-PHA was extracted with ethyl acetate. The organic phase was filtered away from the remaining biomass and processed further to obtain pure PHA polymers. The spent biomass was dried in a laminar oven for 24–48 h at 60 °C to remove solvent residues. In this manner, two spent kinds of biomass containing no polymers were obtained: ZDR, originating from the *Z. denitrificans* P(3HB) process, and PPR, biomass from *P. putida* mcl-PHA fermentation, and both were used in further studies.

### 3.4. Biomass Characterization

The dry weight of the spent biomass and the mineral content were determined using the gravimetric method by heating at 105 °C or 550 °C under an oxidative atmosphere, respectively. The total nitrogen and phosphorus contents were determined using Kjeldahl (PN-EN 13342:2002) and the spectrophotometric method (PN-EN ISO 6878:2006), respectively. The water-soluble and acetone-soluble fractions were estimated by extraction of 5 g of biomass placed in a Falcon tube by two 30 mL portions of appropriate solvent, followed by vortexing for 10 min. at r.t. and centrifugation. The solutions were combined and evaporated to dryness in a rotary evaporator.

Amino acid content determination was performed by the automatic amino acid analyzer AAA 400 Ingos (Prague, Czech Republic). Briefly, the biomass samples were hydrolyzed with 6 M HCl at 110 °C for 20 h. Determination of sulfur amino acids was preceded by oxidation of the sample with performic acid, followed by acidic hydrolysis, whereas for the determination of tryptophan, alkaline hydrolysis was applied. Amino acids were separated by ion exchange chromatography using an Ostion LG ANB column (sodium form) at 60 °C or 74 °C and quantified by post-column derivatization with ninhydrin coupled with UV–VIS detection at 440 nm for proline and 570 nm for other amino acids.

Point of zero charge (PZC) values for the used materials were determined with the use of the pH drift method. Using 0.1 M HCl and 0.1 M NaOH, 0.01 M solution of NaCl in 100 cm^3^ Erlenmayer flasks was adjusted to pH values ranging from 2 to 11 and described as the initial pH. In addition, 50 mg of ZDR and PPR was added to different flasks and shaken for 48 h in an orbital shaker for 150 rpm. The pH of the samples was obtained after a specified time. In the next step, the values were plotted against the initial pH. The initial pH at which the change in measured pH was zero was the pH recorded as the PZC point.

### 3.5. Sorption Process

The removal of selected metal ions was carried out in a dynamic batch system. The chosen process parameters that were regulated were the heavy metal concentration and the time of the sorption process.

The adsorption process was carried out in containers with a volume equal to 60 cm^3^. Approximately 0.1 g of ZDR, PPR, ZDE and PPE was used for each experiment. A 40 cm^3^ solution of Cd and Pb with concentrations of 100, 250, 500, 750 and 1000 ppm was added to the sorbent, respectively, and put in a rotary shaker (100 rpm) for the specified times. The pH was set to 5.5 in all of the experiment variants. The removal process was carried out for 5, 10, 15, 20, 30, 60, 90 and 120 min. After the chosen times, the probes were filtered and the filtrate was measured with atomic absorption spectrometry for the presence of residual metal ions. Tests were carried out in triplicates and the results were averaged (the mean standard error did not exceed 5%).

### 3.6. Sorption Equilibrium

The equations used for the calculation of sorption and percentage metal ion removal at equilibrium were used to fit the equilibrium isotherms such as Langmuir, Freundlich and Temkin models. The linearized version of the used models is presented in Table 5.

### 3.7. Sorption Kinetic Modelling

The calculation of kinetics presents important information on the mechanism and rate of adsorption. In this study, kinetic modelling of adsorption was described using pseudo-first (PFO), pseudo-second (PSO) and Weber–Morris models (W-M). The models mentioned above were studied by changing the contact time of the Pb and Cd adsorption on biomass at 5, 10, 15, 30, 60, 90 and 120 min. The linearized forms of the used models are presented in Table 6.

### 3.8. Desorption Studies

The reusability of materials as sorbents is a very important parameter; thus, sorption/desorption studies have been carried out in three cycles. In addition, 0.1 M of organic acids such as acetic acid, citric acid and 0.1 M of sodium chloride, potassium chloride and deionized water have been used as eluents. In the first stage, Cd and Pb ion sorption processes were conducted in 250 cm^3^ containers with the addition of 1 g of ZDE, PPE, ZDR and PPR, respectively, and 100 cm^3^ of prepared metal ion aqueous solutions (500 mg/dm^3^). After 24 h, the residual solution was removed and the filtrate was transferred to another container. In the next stage, the biomass was treated with the chosen eluents for 30 min. After the specified time, the ion concentrations were measured with the use of atomic absorption spectrometry. Using the equation presented below, the degree of desorption was calculated.

(1) $$R_{des}=\frac{M_{sol}}{M_{sor}}\cdot 100\%$$

where *M_sol_*—metal content in solution after desorption (mg); *M_sor_*—metal content in sorbent after desorption (mg).

## 4. Conclusions

In this study, we have shown the possible directions for the utilization of biowaste after PHA pilot-scale production. We have focused on upcycling spent biomass for heavy metal remediation applications. The sorption capacity of the investigated post-processing materials varied from ~100 to 120 mg/g for Cd ions and ~100 to 150 mg/g for Pb ions, indicating that ZD and PP biowastes are efficient adsorbents for the removal of toxic metal cations. The mentioned adsorption capacities for Cd (II) and Pb (II) ions are c.a. three times higher than determined before for some inorganic adsorbents or lignocellulosic materials. The equilibrium adsorption isotherm studies show that the Freundlich isotherm demonstrates the best fit for the removal of chosen metal ions according to the correlation coefficient R^2^. Additionally, kinetic studies proved that the adsorption process is of chemical nature, which is connected to the highest correlation with the pseudo-second-order model. FTIR analysis underlines the participation of different chemical groups in the adsorption process. In conclusion, the presented research broadens the possibilities for the management of bio-waste after the production of PHAs and contributes to improving the technology for the production of these promising biopolymers.

## Figures and Tables

**Table 1:** Composition of the investigated microbial biomass.

Spent Biomass	Dry Weight, %	Mineral Content, %	Total Nitrogen, % d.w.	Total Phosphorus, % d.w.	Water Soluble Fraction, %	Acetone Soluble Fraction, %
ZDR	92.0	9.55	8.6	1.2	31	6
PPR	93.3	9.55	10.1	2.0	22	4

**Table 2:** Results obtained for the Freundlich isotherm model for Cd^2+^ and Pb^2+^.

	Cd^2+^				Pb^2+^			
Parameters	ZDR	ZDE	PPR	PPE	ZDR	ZDE	PPR	PPE
Freundlich								
R^2^	0.9961	0.9784	0.9856	0.9942	0.9967	0.9794	0.9822	0.9808
1/n	0.9977	0.9280	0.8094	0.7408	0.2595	0.6436	0.5791	0.6197
K_F_(mg^1−(1/n)^(dm^3^)^1/n^g^−1^)	0.3166	0.3212	0.7314	1.1972	38.587	2.5852	4.1795	3.0740

**Table 3:** Results obtained for the pseudo-second kinetic model.

**parameter**	**ZDE**					**ZDR**					**PPE**					**PPR**				
	**Cd^2+^ Concentration (mg/dm^3^)**																			
	100	250	500	750	1000	100	250	500	750	1000	100	250	500	750	1000	100	250	500	750	1000
**II Order**																				
**q_e_**	14.779	34.571	72.875	115.323	111.936	12.921	29.020	58.236	93.899	117.836	16.374	31.291	65.540	104.629	101.982	38.901	31.089	108.334	125.343	101.789
**k_2_**	0.0019	0.0007	0.0004	0.0002	0.0005	0.0093	0.0046	0.0032	0.0012	0.0014	0.0012	0.0024	0.0006	0.0005	0.0009	0.0001	0.0017	0.0001	0.0002	0.0007
**R^2^**	0.983	0.957	0.890	0.985	0.992	0.999	0.995	0.995	0.998	0.998	0.940	0.989	0.973	0.969	0.994	0.971	0.984	0.796	0.853	0.996
**parameter**	**ZDE**					**ZDR**					**PPE**					**PPR**				
	**Pb^2+^ Concentration (mg/dm^3^)**																			
	100	250	500	750	1000	100	250	500	750	1000	100	250	500	750	1000	100	250	500	750	1000
**II Order**																				
**q_e_**	14.710	76.371	91.155	103.421	147.170	21.9483	51.6364	98.5165	116.8087	140.1165	19.3292	61.1734	95.8474	103.6422	179.2039	15.0507	54.9285	83.0469	103.5272	145.8427
**k_2_**	0.0027	0.0002	0.0004	0.0009	0.0002	0.0053	0.0042	0.0018	0.0015	0.0008	0.0010	0.0003	0.0003	0.0009	0.0001	0.0028	0.0005	0.0007	0.0012	0.0002
**R^2^**	0.9332	0.9702	0.9772	0.9897	0.9855	0.9972	0.9992	0.9996	0.9960	0.9905	0.9530	0.9840	0.9683	0.9943	0.9541	0.9291	0.9849	0.9965	0.9953	0.9914

**Table 4:** Desorption results.

Elution (%)			
Eluent	Cycle 1	Cycle 2	Cycle 3
**ZDR**			
Acetic acid	74	68	77
Citric acid	85	91	86
Potassium chloride	24	31	19
Sodium chloride	28	25	27
Deionized water	0.4	0.2	0.7
**ZDE**			
Acetic acid	69	71	63
Citric acid	79	88	76
Potassium chloride	19	16	18
Sodium chloride	22	25	27
Deionized water	0.8	0.2	0.7
**PPR**			
Acetic acid	81	78	74
Citric acid	92	87	89
Potassium chloride	17	24	21
Sodium chloride	22	25	17
Deionized water	0.8	0.5	0.1
**PPE**			
Acetic acid	78	75	72
Citric acid	95	91	93
Potassium chloride	28	24	18
Sodium chloride	28	22	26
Deionized water	0.2	0.4	0.3

**Table 5:** Linearized equation of Langmuir, Freundlich and Temkin isotherm models.

Model	Equation
Langmuir	$\frac{C_{e}}{q_{e}}=\frac{C_{e}}{q_{\max}}+\frac{1}{b\cdot q_{\max}{}_{}}$
Freundlich	${\log\;q}_{e}={\mathrm{logK}}_{f}+\frac{1}{n}{\log\;C}_{e}$
Temkin	$q_{e}$= B ln $K_{t}$+ B ln $C_{e}$ B = $\frac{\mathrm{RT}}{b_{t}}$

where C_e_—metal ion concentration in equilibrium (mg/dm^3^); q_e_—sorption capacity in equilibrium (mg/g); q_max_—maximum sorption capacity (mg/g); b—Langmuir constant (dm^3^/mg); K_f_—Freundlich constant (mg^1−(1/n)^(dm^3^)^1/n^g^−1^); n—heterogeneity coefficient; K_t_—constant binding equilibrium responsible for the maximum binding energy (dm^3^/g); B—constant associated with the sorption heat (J/mol); R—gas constant (8.314 J mol/K); T—temperature (K); b_t_—Temkin isotherm constant.

**Table 6:** Sorption kinetics models.

Model	Equation
PFO	log ($q_{e}$−$q_{t})={\log\;q}_{e}-\frac{k_{1}}{2303}$
PSO	$\frac{t}{q_{t}}=\frac{1}{k_{2}q_{e}^{2}}+\frac{t}{q_{e}}$
W-M	$q_{t}$= $K_{\mathrm{id}}{\;t}^{0.5}+\;I$

where k_1_—pseudo-first-order kinetics constant (1/min); k_2_—pseudo-second-order kinetics constant; k_id_—the intra-particle diffusion rate constant (mg/g min^0.5^); I—intercept of the line in the Weber–Morris model.

## Data Availability

The data presented in this study is available in the article and Supplementary Materials.

## Supplementary Materials

The following supporting information can be downloaded at: https://www.mdpi.com/article/10.3390/molecules28176345/s1, Figure S1: H-NMR spectra (50 mg/mL in DMSO-d6) of acetone-soluble fraction extracted from the spent biomass ZDR; Figure S2: H-NMR spectra (50 mg/mL in CDCl_3_) of acetone-soluble fraction extracted from the spent biomass PPR; Figure S3: FTIR spectra of acetone-soluble fraction extracted from the spent biomass (A) ZDR; (B) PPR; Figure S4: The graphs of the Langmuir and Temkin isotherm models for both Cd2+ and Pb2 for all used biomass; Figure S5: The graphs of the PFO and W-B kinetic models for all used concentrations 100, 250, 500, 750, and 1000 ppm of both Cd2+ and Pb2+ for ZDE, ZDR, PPE and PPR biomass, respectively Table S1: Amino acids profile (mg/g) of the spent biomass ZDR and PPR; Table S2: Parameters for Langmuir, Freundlich and Temkin isotherm models, Table S3: Parameters for pseudo-first-rate order model, pseudo-second-rate order model and Weber- Morris kinetic model.
