# Supplementary material for: Upcycling Waste Streams from a Biorefinery Process—A Case Study on Cadmium and Lead Biosorption by Two Types of Biopolymer Post-Extraction Biomass
Source: Molecules. 2023 Aug 30;28(17):6345. doi: 10.3390/molecules28176345 (PMC10488894; doi:10.3390/molecules28176345)
Supplement: Supplementary file 1 [file molecules-28-06345-s001.zip › molecules-2554010-supplementary.pdf]

Supplementary Materials

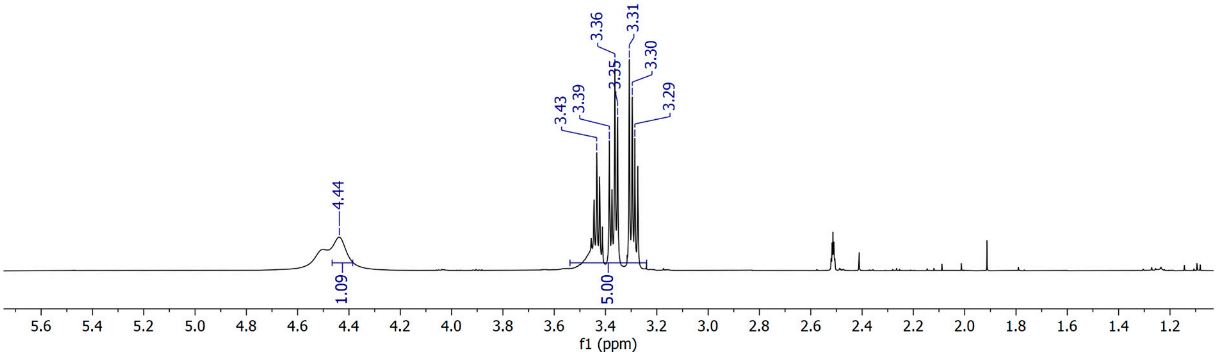

Figure S1. ^{1}H-NMR spectra (50 mg/ml in DMSO-d6) of acetone-soluble fraction extracted from the spent biomass ZDR.

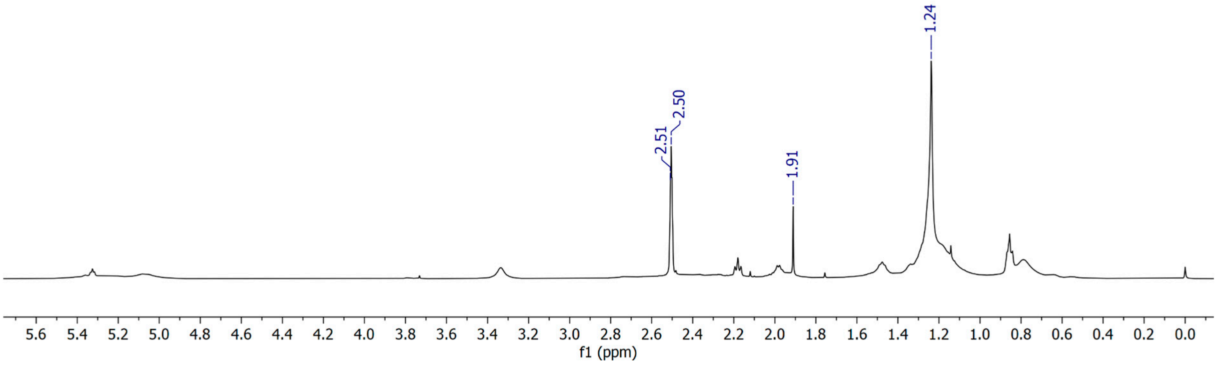

Figure S2. ^{1}H-NMR spectra (50 mg/ml in $CDCl_3$) of acetone-soluble fraction extracted from the spent biomass PPR.

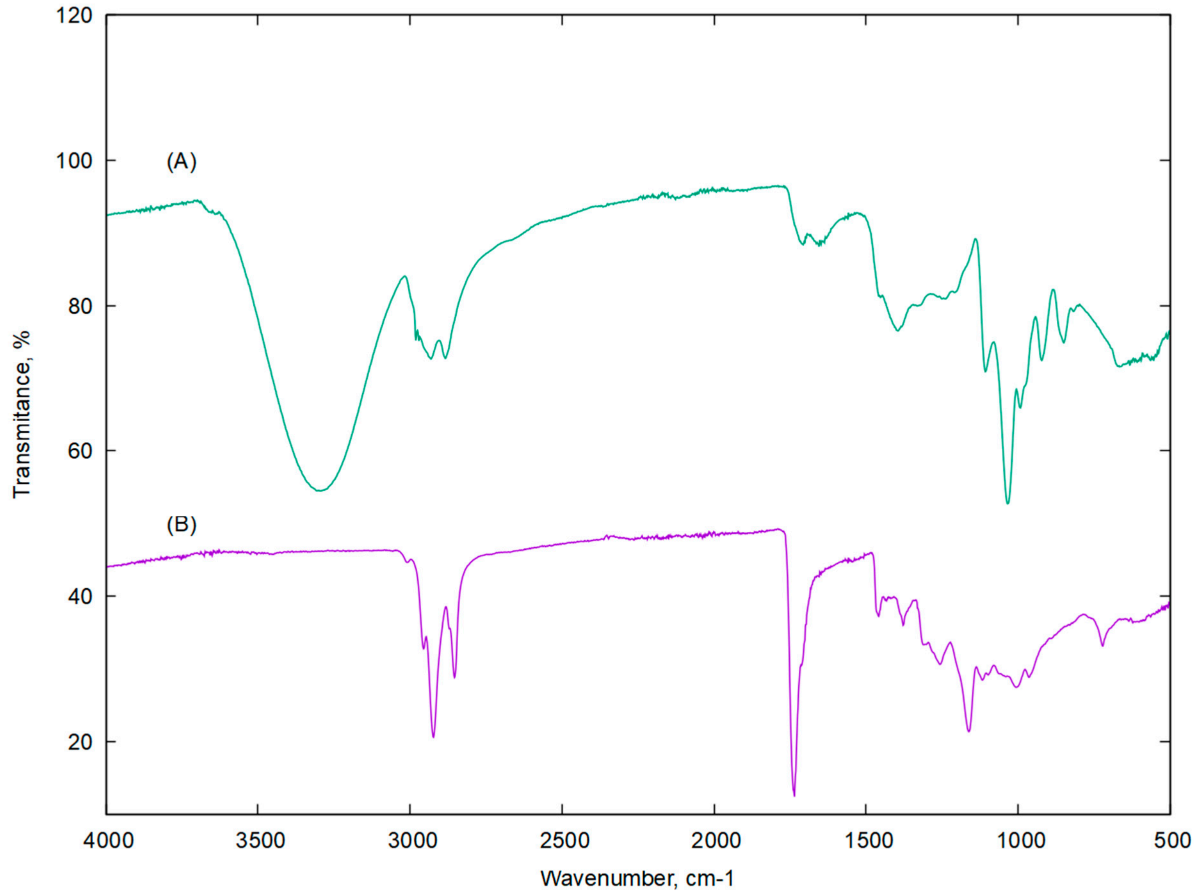

Figure S3. FTIR spectra of acetone-soluble fraction extracted from the spent biomass (A) ZDR; (B) PPR.

Table S1. Amino acids profile (mg/g) of the spent biomass ZDR and PPR

Amino acid	ZDR	PPR
Asp	52.0	48.2
Thr	25.8	25.3
Ser	18.9	18.3
Glu	63.5	72.3
Pro	18.2	19.7
Gly	27.1	28.1
Ala	38.4	41.2
Cys. acid	7.90	5.39
Val	29.4	29.4

Sulf met	26.5	13.0
Ile	24.3	19.8
Leu	43.5	42.8
Tyr	15.7	15.7
Phe	20.2	20.3
His	12.7	11.0
Lys	27.7	34.7
Arg	32.3	32.7
Trp	17.5	17.7

Table S2. Parameters for Langmuir, Freundlich and Temkin isotherm models

	**Cd^{2+}**				**Pb^{2+}**			
**Parameters**	**ZDR**	**ZDE**	**PPR**	**PPE**	**ZDR**	**ZDE**	**PPR**	**PPE**
**Langmuir**								
**R^2**	0.3115	0.1704	0.8763	0.9286	0.9900	0.8798	0.8640	0.8707
**q_{max} (mg/g)**	213.33	667.90	231.76	202.35	153.18	173.49	172.10	173.85
**K_l (dm^3/g)**	0.0025	0.0004	0.0016	0.0023	0.1102	0.0046	0.0064	0.0051
**Freundlich**								
**R^2**	**0.9961**	**0.9784**	**0.9856**	**0.9942**	**0.9967**	**0.9794**	**0.9822**	**0.9808**
**1/n**	0.9977	0.9280	0.8094	0.7408	0.2595	0.6436	0.5791	0.6197
**K_F($mg^{1-(1/n)}(dm^3)^{1/n}g^{-1}$)**	0.3166	0.3212	0.7314	1.1972	38.587	2.5852	4.1795	3.0740
**Temkin**								
**R^2**	0.9328	0.7714	0.9439	0.9357	0.9557	0.9078	0.8829	0.8930
**K_T (dm^3/g)**	0.0255	0.0502	0.0318	0.0405	18.1928	0.0686	0.1056	0.0793
**B_T**	52.455	94.8358	70.7432	72.4380	144.927	74.5844	78.0974	75.8684

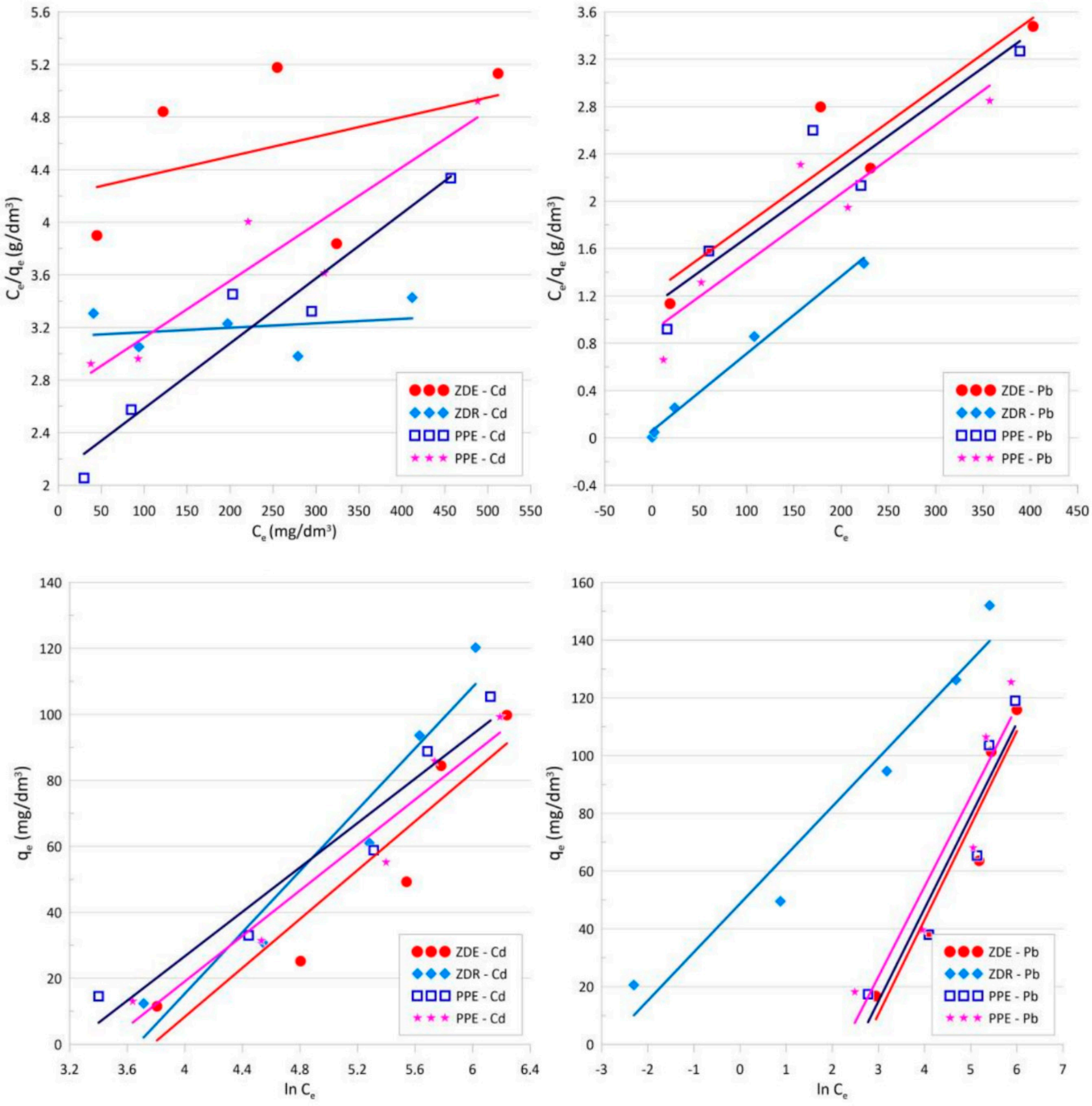

Figure S4. The graphs of the Langmuir and Temkin isotherm models for both Cd2+ and Pb2 for all used biomass.

Table S3. Parameters for pseudo-first-rate order model, pseudo-second-rate order model and Weber- Morris kinetic model

parameter	**ZDE**					**ZDR**					**PPE**					**PPR**				
	**Cd^{2+} Concentration (mg/dm^3)**																			
	**100**	**250**	**500**	**750**	**1000**	**100**	**250**	**500**	**750**	**1000**	**100**	**250**	**500**	**750**	**1000**	**100**	**250**	**500**	**750**	**1000**
**I order**																				

parameter	ZDE					ZDR					PPE					PPR				
	**Cd^{2+} Concentration (mg/dm^3)**																			
	**100**	**250**	**500**	**750**	**1000**	**100**	**250**	**500**	**750**	**1000**	**100**	**250**	**500**	**750**	**1000**	**100**	**250**	**500**	**750**	**1000**
**q_e**	12.25	25.04	46.71	88.20	94.89	9.236	20.36	38.36	67.68	75.61	14.04	26.80	58.32	77.36	83.92	13.08	27.96	59.14	78.51	82.91
**k1**	0.039	0.033	0.053	0.035	0.043	0.050	0.032	0.049	0.039	0.044	0.015	0.029	0.037	0.045	0.034	0.012	0.030	0.037	0.043	0.036
**R^2**	0.943	0.941	0.811	0.956	0.944	0.980	0.958	0.782	0.987	0.920	0.999	0.977	0.923	0.940	0.972	0.963	0.961	0.936	0.923	0.975
**II order**																				
**q_e**	14.779	34.571	72.875	115.323	111.936	12.921	29.020	58.236	93.899	117.836	16.374	31.291	65.540	104.629	101.982	38.901	31.089	108.334	125.343	101.789
**k2**	0.0019	0.0007	0.0004	0.0002	0.0005	0.0093	0.0046	0.0032	0.0012	0.0014	0.0012	0.0024	0.0006	0.0005	0.0009	0.0001	0.0017	0.0001	0.0002	0.0007
**R^2**	**0.983**	**0.957**	**0.890**	**0.985**	**0.992**	**0.999**	**0.995**	**0.995**	**0.998**	**0.998**	**0.940**	**0.989**	**0.973**	**0.969**	**0.994**	**0.971**	**0.984**	**0.796**	**0.853**	**0.996**
**Weber Morris**																				
**I**	-0.	-1.	1.891	-8.	3.626	3.383	8.642	21.593	23.040	43.421	-1.5	3.5	-1.0	7.7	15.6	-2.3	1.1	-5.8	3.5	10.3

parameter	ZDE					ZDR					PPE					PPR				
	**Cd^{2+} Concentration (mg/dm^3)**																			
	**100**	**250**	**500**	**750**	**1000**	**100**	**250**	**500**	**750**	**1000**	**100**	**250**	**500**	**750**	**1000**	**100**	**250**	**500**	**750**	**1000**
	920	597		550							94	80	07	35	25	86	35	85	13	42
**K_{id}**	1.378	2.960	5.838	10.115	11.223	1.102	2.368	4.671	8.092	8.978	1.359	3.018	6.358	9.687	9.712	1.223	3.081	6.536	9.479	9.970
**R^2**	0.946	0.952	0.739	0.962	0.903	0.900	0.958	0.756	0.945	0.900	0.990	0.963	0.919	0.816	0.939	0.987	0.946	0.931	0.826	0.926

parameter	ZDE					ZDR					PPE					PPR				
	**Pb^{2+} Concentration (mg/dm^3)**																			
	**100**	**250**	**500**	**750**	**1000**	**100**	**250**	**500**	**750**	**1000**	**100**	**250**	**500**	**750**	**1000**	**100**	**250**	**500**	**750**	**1000**
**I order**																				
**q_e**	15.639	42.758	67.364	79.694	103.081	11.3755	26.7088	56.7280	75.7642	102.0885	16.7100	41.8611	63.2710	79.3415	111.4917	16.6684	41.5017	62.4478	73.0049	110.5591
**k1**	0.0206	0.0390	0.0607	0.0466	0.0227	0.0277	0.0666	0.0584	0.0340	0.0238	0.0162	0.0405	0.0502	0.0401	0.0279	0.0180	0.0421	0.0513	0.0392	0.0243

parameter	ZDE					ZDR					PPE					PPR				
	**Pb^{2+} Concentration (mg/dm^3)**																			
	**100**	**250**	**500**	**750**	**1000**	**100**	**250**	**500**	**750**	**1000**	**100**	**250**	**500**	**750**	**1000**	**100**	**250**	**500**	**750**	**1000**
**R_2**	0.8751	0.9792	0.9869	0.8482	0.9520	0.9715	0.9470	0.9275	0.9622	0.9835	0.9728	0.9630	0.9949	0.9149	0.9823	0.9358	0.9925	0.9934	0.9116	0.9842
**II order**																				
**q_e**	14.710	76.371	91.155	103.421	147.170	21.9483	51.6364	98.5165	116.8087	140.1165	19.3292	61.1734	95.8474	103.6422	179.2039	15.0507	54.9285	83.0469	103.5272	145.8427
**k2**	0.0027	0.0002	0.0004	0.0009	0.0002	0.0053	0.0042	0.0018	0.0015	0.0008	0.0010	0.0003	0.0003	0.0009	0.0001	0.0028	0.0005	0.0007	0.0012	0.0002
**R_2**	**0.9332**	**0.9702**	**0.9772**	**0.9897**	**0.9855**	**0.9972**	**0.9992**	**0.9996**	**0.9960**	**0.9905**	**0.9530**	**0.9840**	**0.9683**	**0.9943**	**0.9541**	**0.9291**	**0.9849**	**0.9965**	**0.9953**	**0.9914**
**Weber Morris**																				
**I**	-0.3223	-7.6321	-0.6130	19.0121	-8.3890	6.2569	21.9045	36.4522	44.7039	33.9492	-1.7824	-5.7103	-0.7674	18.8547	-10.9293	-0.2304	-3.3769	4.2936	27.6955	-4.7580
**K_{id}**	1.4320	5.4307	8.5561	9.7597	13.3430	1.7567	3.8177	7.7236	8.7328	11.8880	1.6054	5.1547	8.5993	9.7502	14.35	1.4738	5.1428	8.2123	9.0299	13.72

parameter	ZDE					ZDR					PPE					PPR				
	Pb^{2+} Concentration (mg/dm^3)																			
	100	250	500	750	1000	100	250	500	750	1000	100	250	500	750	1000	100	250	500	750	1000
															22					73
R^2	0.9775	0.9368	0.8209	0.7879	0.9694	0.9118	0.6333	0.7440	0.9360	0.9715	0.9952	0.9349	0.8441	0.8666	0.9572	0.9872	0.9278	0.8606	0.8614	0.9737

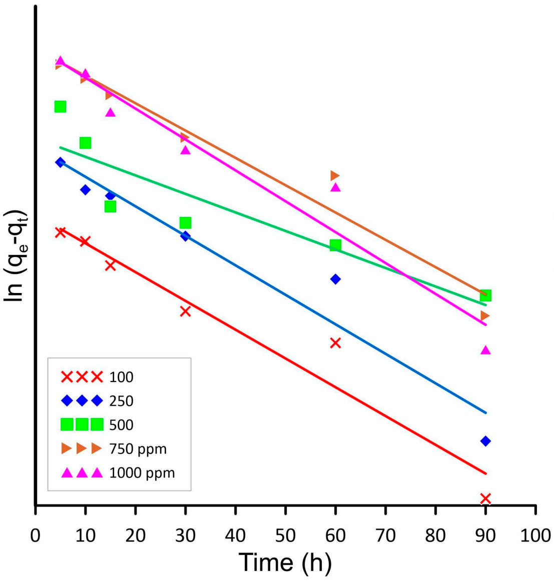

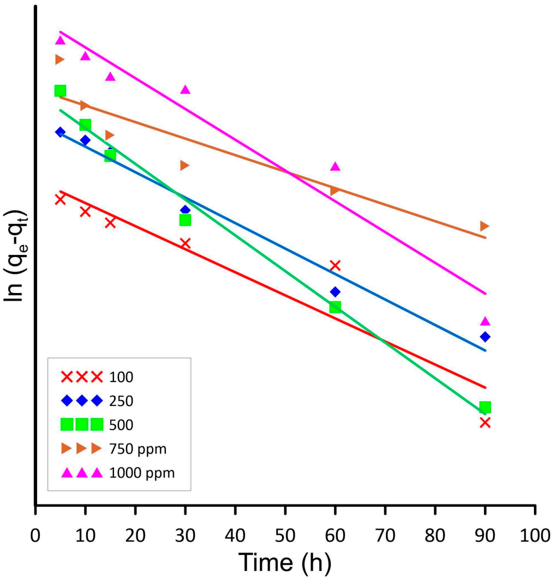

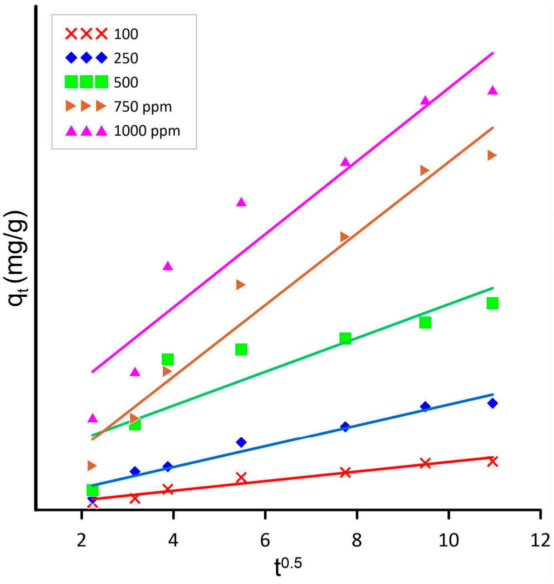

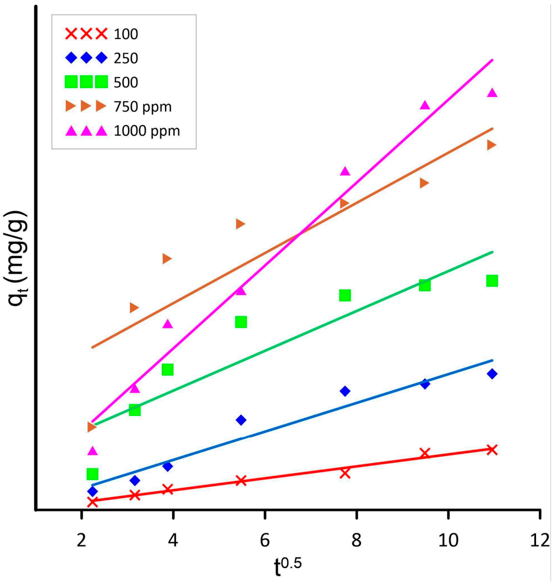

ZDE

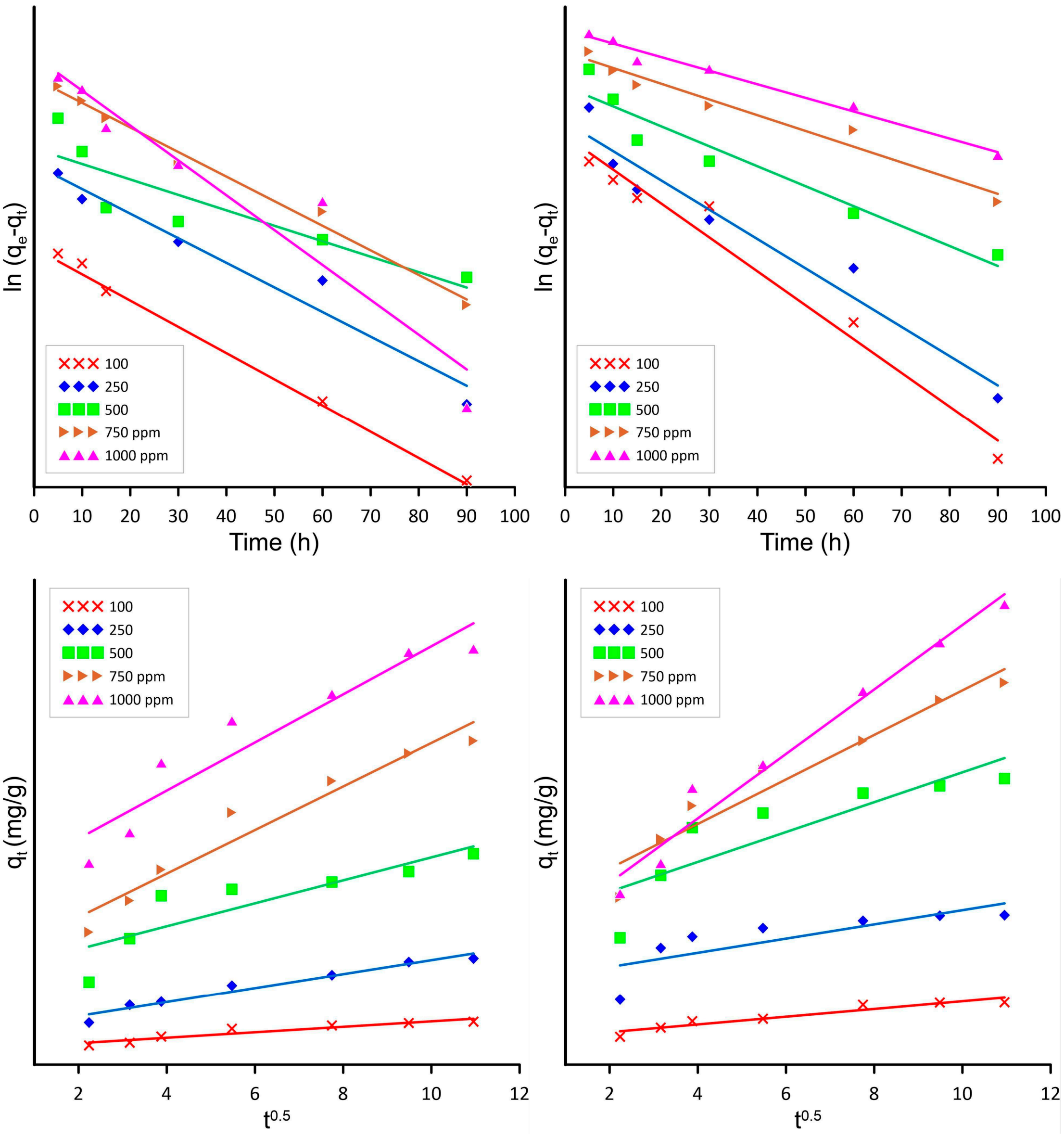

ln (q_e-q_t)
Time (h)
q_t (mg/g)
$t^{0.5}$
100
250
500
750 ppm
1000 ppm

ZDR

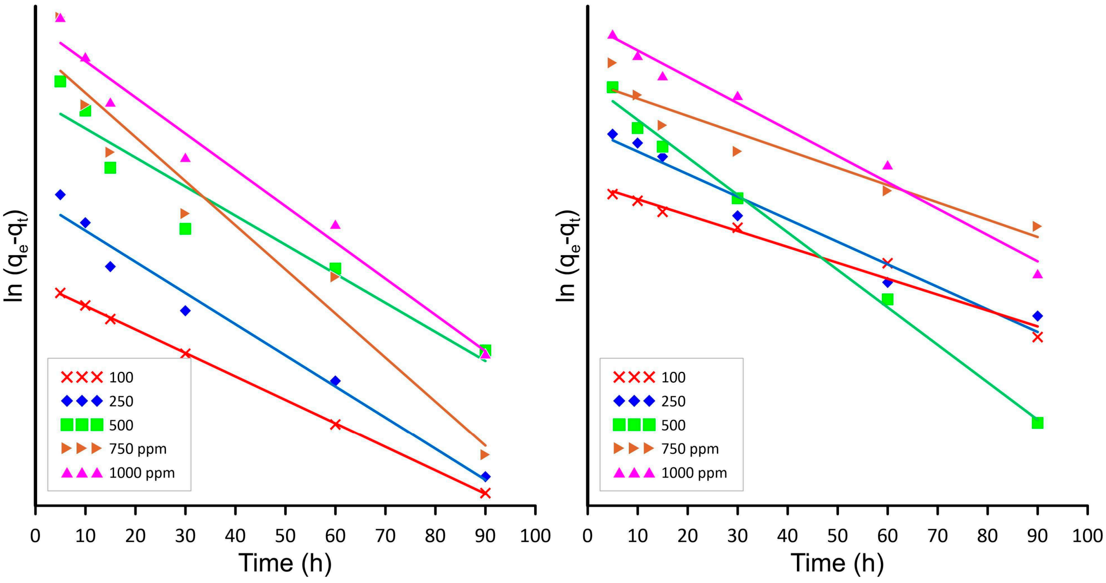

ln (q_e-q_t)
Time (h)
100
250
500
750 ppm
1000 ppm

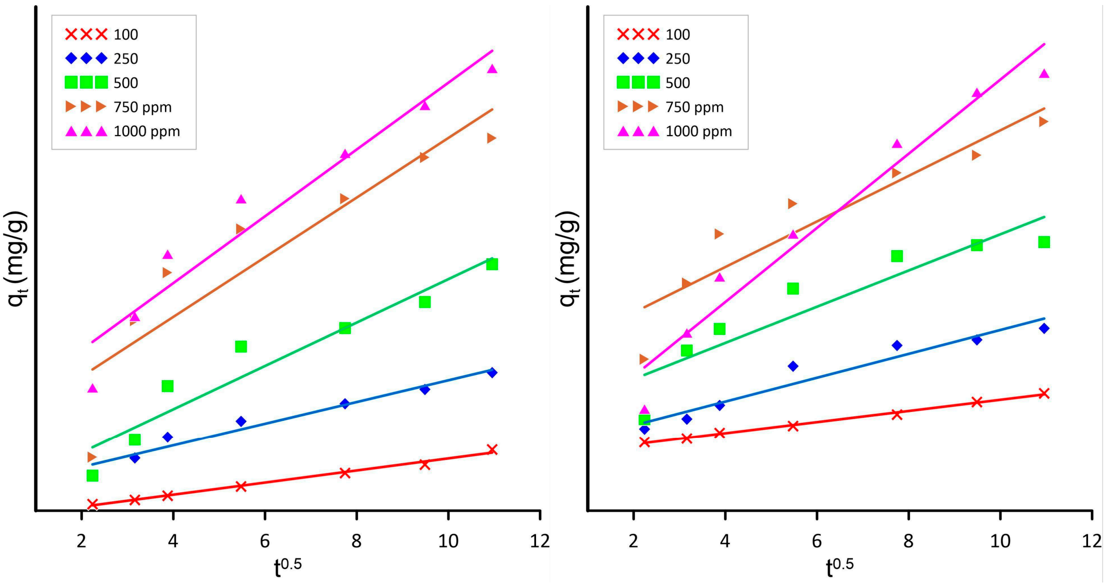
100
250
500
750 ppm
1000 ppm
qt (mg/g)
t0.5
2
4
6
8
10
12

PPE

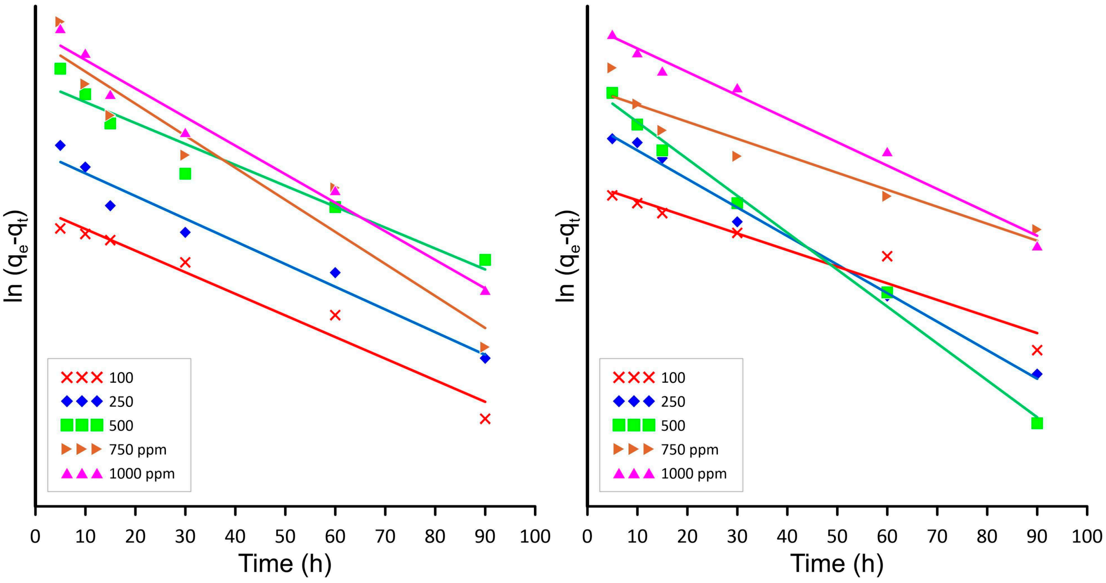
100
250
500
750 ppm
1000 ppm
ln (qe-qt)
Time (h)
0
10
20
30
40
50
60
70
80
90
100

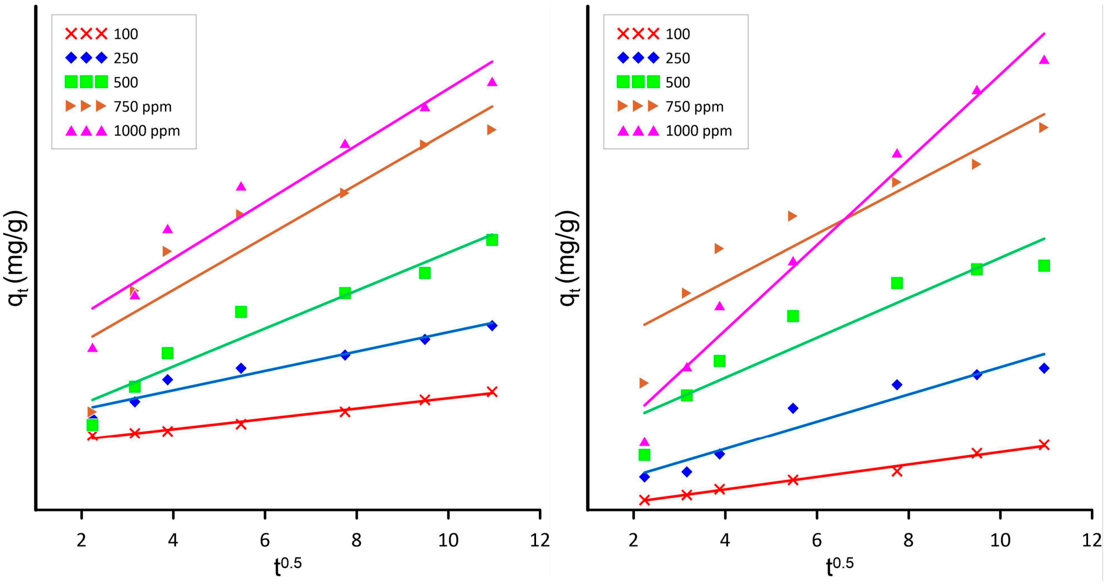
100
250
500
750 ppm
1000 ppm
qt (mg/g)
t0.5
2
4
6
8
10
12

PPR

Figure S5. The graphs of the PFO and W-B kinetic models for all used concentrations 100, 250, 500, 750, and 1000 ppm of both Cd^{2+} and Pb^{2+} for ZDE, ZDR, PPE and PPR biomass, respectively
